# A Common CNR1 (Cannabinoid Receptor 1) Haplotype Attenuates the Decrease in HDL Cholesterol That Typically Accompanies Weight Gain

**DOI:** 10.1371/journal.pone.0015779

**Published:** 2010-12-31

**Authors:** Qiping Feng, Lan Jiang, Richard L. Berg, Melissa Antonik, Erin MacKinney, Jennifer Gunnell-Santoro, Catherine A. McCarty, Russell A. Wilke

**Affiliations:** 1 Division of Clinical Pharmacology, Department of Medicine, Vanderbilt University Medical Center, Nashville, Tennessee, United States of America; 2 Department of Molecular Physiology and Biophysics, Center for Human Genetics Research, Vanderbilt University Medical Center, Nashville, Tennessee, United States of America; 3 Biomedical Informatics Research Center, Marshfield Clinic Research Foundation, Marshfield, Wisconsin, United States of America; 4 Human and Molecular Genetics Center, Medical College of Wisconsin, Milwaukee, Wisconsin, United States of America; 5 Center for Human Genetics, Marshfield Clinic Research Foundation, Marshfield, Wisconsin, United States of America; Dr. Margarete Fischer-Bosch Institute of Clinical Pharmacology, Germany

## Abstract

We have previously shown that genetic variability in CNR1 is associated with low HDL dyslipidemia in a multigenerational obesity study cohort of Northern European descent (209 families, median  = 10 individuals per pedigree). In order to assess the impact of CNR1 variability on the development of dyslipidemia in the community, we genotyped this locus in all subjects with class III obesity (body mass index >40 kg/m^2^) participating in a population-based biobank of similar ancestry. Twenty-two haplotype tagging SNPs, capturing the entire CNR1 gene locus plus 15 kb upstream and 5 kb downstream, were genotyped and tested for association with clinical lipid data. This biobank contains data from 645 morbidly obese study subjects. In these subjects, a common CNR1 haplotype (H3, frequency 21.1%) is associated with fasting TG and HDL cholesterol levels (p = 0.031 for logTG; p = 0.038 for HDL-C; p = 0.00376 for log[TG/HDL-C]). The strength of this relationship increases when the data are adjusted for age, gender, body mass index, diet and physical activity. Mean TG levels were 160±70, 155±70, and 120±60 mg/dL for subjects with 0, 1, and 2 copies of the H3 haplotype. Mean HDL-C levels were 45±10, 47±10, and 48±9 mg/dL, respectively. The H3 CNR1 haplotype appears to exert a protective effect against development of obesity-related dyslipidemia.

## Introduction

Clinical lipid disorders have enormous public health significance. Circulating low density lipoprotein cholesterol (LDL-C) levels are strongly correlated with cardiovascular disease, and pharmacological intervention targeted at reducing LDL-C can markedly reduce risk [1]. High density lipoprotein cholesterol (HDL-C) and fasting triglyceride (TG) levels are also strong predictors of cardiovascular disease [2], [3]. Data obtained from many diverse sources suggest that HDL-C may in fact be cardioprotective. Each 1 mg/dL increase in HDL-C is associated with a 6% reduction in cardiovascular events [2]. The decrease in HDL-C level that typically accompanies weight gain is therefore of considerable importance, particularly in the context of the growing obesity epidemic.

Body mass index (BMI) is inversely correlated with HDL-C level, and genetic variability in endocannabinergic signaling clearly influences both of these traits [4], [5], [6]. We have previously shown that a functional variant (a nonsynonymous coding SNP) in fatty acid amide hydrolase (*FAAH*) is associated with large changes in circulating HDL-C levels (mean HDL-C = 40.5±14.7 mg/dL, 39.1±10.4 mg/dL and 34.8±8.1 mg/dL for subjects with 0, 1, and 2 copies of the variant, p<0.01) [7]. This effect is partly independent of BMI, and insulin responsiveness [7]. The FAAH gene product enzymatically inactivates *N*-arachidonylethanolamine (AEA), the primary endogenous CB1 receptor ligand [8].

Genetic variability in the CB1 receptor itself (gene name *CNR1*) is also associated with dyslipidemia [4], [7]. Our group has previously shown that a common CNR1 haplotype (15% frequency in subjects of Northern European ancestry) is associated with elevated fasting TG levels and reduced levels of HDL-C in one of the most rigorously phenotyped family-based obesity cohorts in the U.S. Again, this effect was partly independent of BMI [4]. Because the relationship between BMI and HDL-C is curvilinear [9], and because the correlation is most pronounced in subjects who are extremely obese [9], we characterized the relationship between dyslipidemia and genetic variability in CNR1 in study subjects with a very high BMI (class III obesity, defined as BMI>40 kg/m^2^).

In the current study, we interrogated the comprehensive electronic medical record of the Marshfield Clinic Personalized Medicine Research Project (n = 20,000), identified all participants with a BMI>40 kg/m^2^, and genotyped 645 of these individuals using 22 haplotype tagging SNPs across the entire CNR1 gene locus (plus 15 kb upstream and 5 kb downstream). To adjust for the potential impact of diet and lifestyle, our analytical models included previously archived data regarding nutrient intake (percent calories from fat, alcohol intake) and physical activity (sport, leisure and occupational activity). We report a CNR1 haplotype (21.1% frequency in subjects of Northern European ancestry) that may protect against the development of low-HDL dyslipidemia during weight gain.

## Methods

### Ethics Statement

The current study was conducted in accordance with the Principles outlined within the Declaration of Helsinki, and all participants have provided informed written consent. Approval was obtained from the Institutional Review Board of the Marshfield Clinic in Wisconsin, and the Institutional Review Board of Vanderbilt University in Tennessee.

### Study Population

This study was conducted using one of the largest population-based biobank cohorts in the U.S., the Marshfield Clinic Personalized Medicine Research Project (PMRP) [10], [11]. The PMRP biobank currently contains DNA and secure, encrypted, electronic medical records for more than 20,000 participants [9], [12]. Subjects within the PMRP biobank were recruited from the surrounding community, a rural population residing in Central Wisconsin [13], [14]. Like the surrounding community, the cohort is 97% non-Hispanic white. The primary ethnic groups include German (78%), Irish (17%), English (16%), Norwegian (12%), Polish (11%), and other Northern European groups. Weight trends in this region closely mirror those of the non-Hispanic white population in the National Health and Nutrition Examination Survey (NHANES).

**Figure 1:**
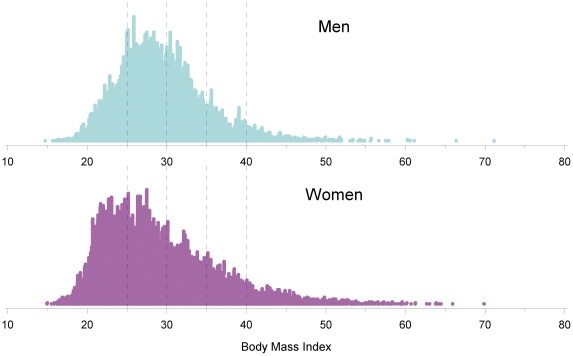
Gender stratified distribution of Body Mass Index (BMI) within the entire population-based PMRP biobank. All subjects with BMI>40 kg/m^2^ (n = 477 female participants, and 168 male participants) were included in the current study cohort.

The gender-stratified distribution of BMI is illustrated in **Figure 1**, for the entire PMRP biobank (n = 20,000). All biobank participants with class III obesity (BMI>40 kg/m^2^) were identified for the current study (n = 645). The current study population is skewed toward females (i.e., 168 male and 477 female participants with BMI>40 kg/m^2^). This female preponderance reflects two factors. First, female patients opted to participate in the PMRP biobank at a slightly higher rate than male patients (58% versus 42%) [10], [15]. Second, within our target community, extreme obesity is more prevalent in females than in males (7.8% versus 4.4%). General subject characteristics are summarized in **Table 1**.

**Table 1:** Patient characteristics.

		Female (N = 477)	Male (N = 168)
		Mean ± SD	Mean ± SD
**Covariates**	**Demographic Information:**		
	**Age (** ***years*** **)**	48.6±14.5	52.1±15.1
	**Weight (** ***lbs*** **)**	263.7±36.6	303.4±36.9
	**Height (** ***inches*** **)**	63.9±3.1	69.4±2.9
	**BMI (** ***kg/m^2^*** **)**	45.4±5.1	44.3±4.2
	**Dietary Intake:**		
	**Total cal. from food (** ***Kcal*** **)**	1763.3±1018.8	2322.4±1281.2
	**Cal. from protein (** ***Kcal*** **)**	71.8±43.3	97.5±59.6
	**Cal. from fat (** ***Kcal*** **)**	65.7±42.9	89.17±55.95
	**Cal. from carbohydrate (** ***Kcal*** **)**	225.4±132.9	275.74±153.69
	**Alcohol consumption (** ***grams*** **)**	3.7±23.2	9.9±20.2
	**Physical Activity:**		
	**Work Index**	2.6±0.7	2.8±0.8
	**Sport Index**	1.8±0.5	1.9±0.5
	**Leisure Index**	2.4±0.6	2.2±0.6
**Endpoints**	**Lipid Phenotypes:**		
	**Median T CHOL (** ***mg/dL*** **)**	193.0±31.2	192.3±31.3
	**Median LDL-C (** ***mg/dL*** **)**	116.7±26.5	116.5±27.5
	**Median HDL-C (** ***mg/dL*** **)**	48.3±10.5	40.9±7.8
	**Median TG (** ***mg/dL*** **)**	151.4±69.2	175.7±75.7

T CHOL: total cholesterol; LDL-C: low density lipoprotein cholesterol; HDL-C: high density lipoprotein cholesterol; TG: triglyceride.

### Phenotyping

All clinical lipid data were electronically extracted from the biobank database, for each of the 645 study subjects. These data reflect longitudinal lipid data collected during the course of routine clinical care, and imported from the comprehensive electronic medical record into the PMRP database. Each lipid trait was then expressed as a median baseline lipid value for every individual. Median lipid levels (Cholesterol, LDL, HDL, and TG) represented our primary endpoints, available on all 645 subjects included in this study.

Because clinical lipid data can be markedly altered by co-morbidity and/or medication use, we have also developed an alternate phenotype, a modeled lipid trait constructed by censoring the longitudinal lipid data for each individual at the first medical record date linked to a relevant co-morbidity (e.g., diabetes mellitus) or lipid modifying medication (e.g., atorvastatin). Our approach has been published [9]. Of the 645 subjects included in the current study, 306 had been exposed to at least one lipid medication during the course of their routine clinical care, 289 (44.8%) had been exposed to statins, 32 (5.0%) to fibric acid derivatives, and 84 (13.0%) to prescription strength niacin. Thus, our secondary traits (i.e., modeled lipid levels) were available for slightly less than half of the cohort (n = 288 with modeled HDL, n = 270 for modeled TGs, and n = 261 for modeled LDL).

### Clinical Covariates

For each PMRP participant, BMI was accurately defined at the time of study entry (**Figure 1**). Height was obtained for each participant using a stadiometer according to standard methods. Weights were also collected at entry, using a beam balance scale according to standard methods. Measurements occurred after the study participant had removed their shoes, hats, and bulky clothing, such as coats and sweaters. Scales were placed in the “zero” position prior to weighing. Weights were recorded to the nearest 1/4 pound (100 grams). BMI was then calculated from the measured height and weight data, by dividing weight in kilograms by height in meters squared [16]. Weights are also available longitudinally, over the course of many years in the clinical notes.

**Figure 2:**
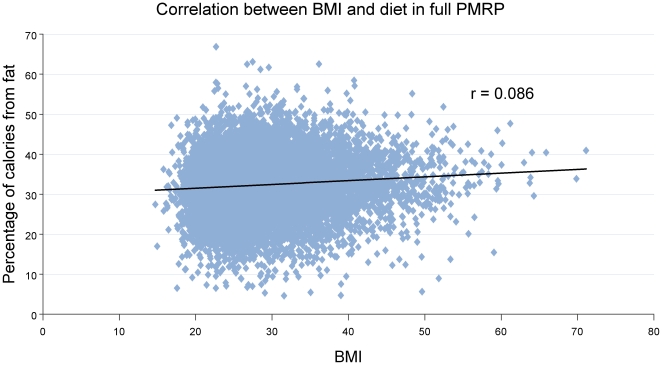
Scatterplot showing the relationship between BMI and dietary fat intake for PMRP participants. Using the entire PMRP biobank** (n = 20,000)**, percentage of daily calories due to fat has been plotted against BMI at the time of study entry. Trend-line is added (r = 0.086).

After enrollment, food frequency questionnaires (FFQs) were used to assess dietary intake. Compared to other epidemiologic methodologies (including weighed food records and 24-hour dietary recalls), FFQs are more representative of usual intake and less expensive to implement, as they are usually self-administered [17], [18]. The selected FFQ for the PMRP, the Diet History Questionnaire (DHQ) (http://riskfactor.cancer.gov/DHQ), was developed by researchers at the National Cancer Institute and has been shown to be superior to the commonly used Willet FFQ and similar to the Block FFQ for estimating absolute nutrient intakes [19], [20], [21], [22], [23], [24], [25]. The DHQ comprises 124 separate food items and asks about portion sizes for most foods. In addition, there are ten questions regarding nutrient supplement intake. The DHQ was printed and scanned by Optimum Solutions (CA, USA). After scanning, the data from the questionnaires were stored in ASCII format and uploaded into a nutrient analysis software package. Diet*Calc software, available from the NIH, was used to convert DHQ data into macronutrient intake [26]. The relationship between BMI and dietary fat intake (% of daily calories due to fat) is shown in **Figure 2**.

Physical activity was also quantified for use in the current study. Standardized surveys are the most practical approach for assessing activity level in large populations. A self-administered physical activity questionnaire, the ARIC/Baecke questionnaire, was mailed to each biobank participant. The ARIC/Baecke questionnaire represents a modified version of the original Baecke Questionnaire [27], comprised of 16 questions, generating three indices of activity: 1) a work index, 2) a sport index, and 3) a leisure-time index [28]. This questionnaire has previously been validated with physiological methods [28].

### Genotyping

**Figure 3:**
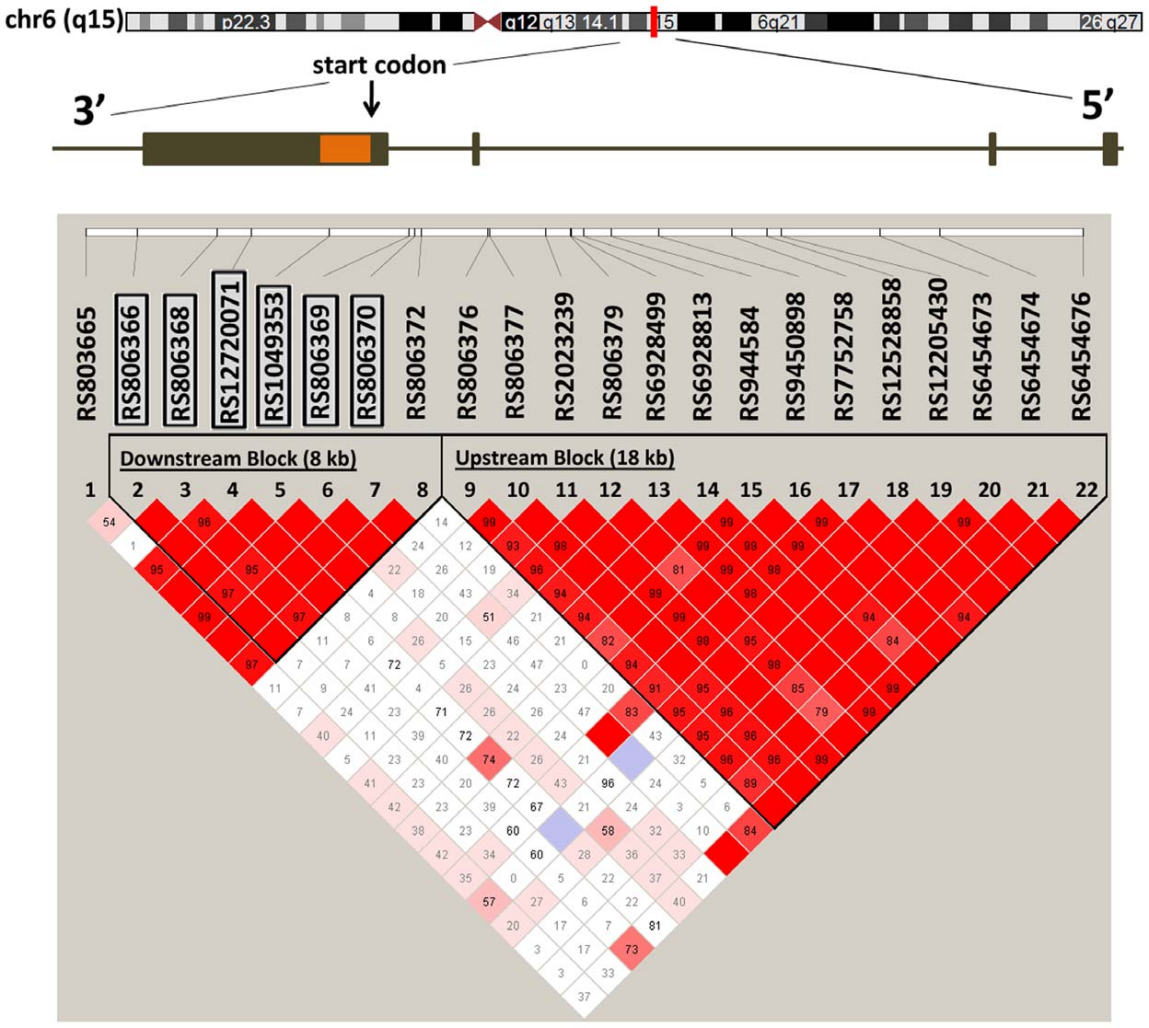
Chromosomal location for CNR1, and linkage disequilibrium (LD) structure within the PMRP database. The chromosome position of the CNR1 gene was obtained from the UCSC human genome browser for the entire gene plus 15 kb upstream and 5 kb downstream (http://genome.ucsc.edu/). SNPs within the identified region were quantified within our study cohort (n = 645) and entered into Haploview for an assessment of LD and tagSNP assignment. Applying the confidence interval method implemented in Haploview version 4.0, we observed two haplotype blocks covering the entire region of interest. CNR1: Cannabinoid receptor 1; LD: Linkage disequilibrium; UCSC: University of California, Santa Cruz.

Haplotype tagging SNPs were identified using our previously published approach [4]. Chromosomal position of the CNR1 gene was obtained from the UCSC human genome browser (http://genome.cse.ucsc.edu/cgi-bin/hgGateway). SNPs within the region of interest were downloaded from the International Human Haplotype Map for the Centre d'Etude du Polymorphisms Humain (CEPH) and entered into Haploview for an analysis of linkage disequilibrium (LD) and tag SNP assignment. The extent of LD across this region was quatified [29] and tag SNPs were identified using Tagger [30]. Cladograms were constructed applying the confidence interval method implemented in Haploview version 4.0 [31]. We have previously applied this approach to successfully identify SNPs associated with dysplipidemia in familes of Northern European descent [4]. The block structure for our region of interest is shown in **Figure 3**, based upon pairwise correlation (D') in our current cohort. This structure is similar to the structure in our prior report [4].

Genotyping was conducted using commercial Taqman Allelic discrimination assays available through Applied Biosystems, Inc. (ABI, Foster City, CA, USA). This method allows direct detection of a thermocycling product by the release of a fluorophor as a result of the Taq polymerase's 5′ exonuclease activity [32]. The individual allele specific oligonucleotide assays are shown in **Table 2**. All assays were performed according to the manufacturer's specifications (ABI): After a run-in period (50.0° for 2 minutes followed by 95.0° for 10 minutes), each reaction was cycled forty times (92.0° for 15 seconds followed by 60.0° for 1 minute) then held at 4.0° until the fluorescence was read by an ABI 7700 Sequence Detector. Each assay contained two different Taqman probes, uniquely labeled to bind to separate (major versus minor) alleles. Each probe consisted of an oligonucleotide with a 5′ reporter dye and 3′ quencher dye. When intact, the proximity of the quencher to the reporter suppresses the fluorescence of the fluorophor. If the probe binds the allele, then taq polymerase cleaves the quencher from the probe, resulting in increased fluorescence. The fluorescent signal was read and quantified by the ABI 7700 Sequence Detector, and the manufacturer-supplied software (ABI) interpreted the relative fluorescent levels of the dye for each sample, calculating the final genotype.

**Table 2:** Tag SNP minor allele frequencies.

SNP	Assay (TaqMan)	MAF^1^ reported	MAF^2^ observed
806365	C__1652594	0.43 (T)	0.40 (T)
**806366**	C___1652593	0.48 (C)	0.48 (C)
**806368**	C___8943804	0.19(C)	0.20 (C)
**12720071**	C___30749291	0.11 (C)	0.09 (G)
**1049353**	C___1652590	0.26(A)	0.31 (A)
**806369**	C___8943784	0.32 (T)	0.27 (T)
**806370**	C___1652589	0.15 (T)	0.09 (T)
806372	C__8943783	0.15 (C)	0.12 (C)
806376	C__1652586	0.43 (C)	0.50 (C)
806377	C__1652585	0.48 (C)	0.48 (T)
2023239	C__11600616	0.17 (C)	0.17 (C)
806379	C__1652584	0.44 (T)	0.48 (T)
6928499	C__30749294	0.18 (C)	0.16 (C)
6928813	C__30749295	0.17 (G)	0.16 (G)
9444584	C__30598869	0.2 (T)	0.18 (T)
9450898	C__30274410	0.16 (T)	0.16 (T)
7752758	C__9863393	0.11 (G)	0.12 (G)
12528858	C__9863392	0.05 (G)	0.07 (G)
12205430	C__30749298	0.19 (C)	0.21 (C)
6454673	C__27392043	0.26 (A)	0.30 (A)
6454674	C__11418433	0.29 (G)	0.31 (G)
6454676	C__28979979	0.08 (A)	0.11 (A)

All SNPs used for haplotype construction are bold.

1 anticipated MAF based on CEPH cohort (abstracted from ABI website/product catalog).

2 observed MAF within 645 PMRP study subjects with Class 3 Obesity (BMI>40 kg/m^2^).

### Statistical Analyses

Genotype-phenotype association tests were conducted using median lipid values as the primary endpoint. The following traits were available on all 645 subjects included in this study: median total cholesterol level, median LDL cholesterol level, median HDL cholesterol level, median TG level, and median TG/HDL-C ratio. Because the TG distribution was skewed, TGs were log transformed. Statistical analyses included tests for association of lipids with single SNPs and haplotypes. All tests involving our primary endpoints were performed using PLINK, a free, open-source genetic analysis toolset (http://pngu.mgh.harvard.edu/purcell/plink/) [33]. This platform was selected based on its efficiency, flexibility and ease of application. The **--freq** option was used to calculate minor allele frequency (MAF), and the **--hardy** option was used to calculate Hardy Weinberg equilibrium (HWE). No SNPs in this study significantly deviated from HWE.

A linear regression model was applied to test the relationship between each SNP and the median lipid traits representing our primary endpoint using the **--linear** option in PLINK. Asymptotic p-values were reported. Initially, 3 inheritance modes were tested: additive, dominant and recessive. An additive model, which is the default mode in PLINK, fit our data best, and an additive model was therefore chosen for all subsequent analyses. We also estimated the influence of environmental factors, dietary intake and physical activity by including them as covariates in the linear regression model using the **--covar** option.

As noted above, modeled lipid traits were also developed for a set of secondary analyses. For these analyses, longitudinal lipid data were right censored at the first mention of a relevant co-morbidity or medication known to modify lipid homeostasis, according to our previously published approach [9]. These secondary traits were available for slightly less than half of the cohort (n = 288 with modeled HDL, n = 270 for modeled TGs, and n = 261 for modeled LDL). In a confirmatory series of tests, they were analyzed for association with single SNPs using a non-parametric approach (Kruskal-Wallis test). When these confirmatory tests localized all SNP associations to the single block of LD characterized in our prior work [4], we proceeded with haplotypes using only that locus.

Haplotypes were estimated using 6 tag SNPs contained within our 15 kb locus of interest, by applying standard E-M algorithms located in the **--hap** option of PLINK. A linear regression model was applied to test each haplotype (vs all other haplotypes), using the **--hap-linear** option. All haplotypes with frequency ≥5% were included in these analyses. Permutation testing was applied to reduce the likelihood that our reported associations were observed simply by chance. Results are reported in both unadjusted and adjusted format (adjusting for age, gender, BMI, physical activity and dietary fat intake).

## Results

A total of 168 male and 477 female biobank participants with class III obesity (BMI>40 kg/m^2^) were included in this study. Of these, 70 males and 162 females were diabetic. Participant characteristics are summarized in **Table 1**, including lipid phenotypes, dietary intake and physical activity. Mean age for male and female participants were 52.1±15.1 and 48.7±14.6 years, respectively. The PMRP claims 97% Northern European ancestry.

Genotyping was performed on 645 DNA samples using the 22 haplotype tagging SNPs listed in **Table 2**. All SNPs followed Hardy-Weinberg (H-W) equilibrium in this cohort, and the observed allele frequencies compared favorably with published frequencies [34].

### Original region of interest

The *CNR1* gene is found on the reverse strand of chromosome 6. As mentioned above, and in our previous report [4], this locus contains two discrete blocks of LD. The coding region is contained in a single exon, located in the downstream block [4]. The second block, proposed to contain additional noncoding exons [35], is located further upstream.

We performed our primary analyses using tagging SNPs from the downstream block and plasma TG level, the trait most strongly associated with CNR1 in our prior work [4]. As illustrated in **Table 3**, rs806372 (a SNP in the putative promoter region of CNR1) shows suggestive association with logTG. As shown, the strength of this association increases when the data are adjusted for dietary intake and physical activity. The strength of the association did not change when the data were further adjusted for age, gender, BMI, carbohydrate intake and alcohol consumption (p = 0.063, n = 578, data not shown). Three inheritance modes were tested (not shown). As in our prior work, an additive mode was the best predictor of association. All subsequent analyses used this mode.

**Table 3:** Replication and refinement of the model (p-values are shown, calculated using a linear regression model in PLINK).

		Log TG [additive] p-value, adjusted by:				
SNP	MAF	Without Adjustment	BMI	Grams EtOH consumption	Percent of kCal from Fat	Sport Index
RS806365	0.402	0.863	0.874	0.946	0.954	0.963
RS806366	0.48	0.109	0.108	0.116	0.107	0.066
RS806368	0.202	0.071	0.071	0.065	0.061	**0.048**
RS12720071	0.086	0.418	0.419	0.382	0.371	0.421
RS1049353	0.312	0.863	0.863	0.788	0.82	0.985
RS806369	0.271	0.447	0.447	0.449	0.46	0.614
RS806370	0.088	0.295	0.299	0.273	0.263	0.192
RS806372	0.122	**0.045**	**0.044**	**0.037**	**0.035**	**0.027**

### Expanded region of interest

The transcription start site for the CNR1 is thought to reside within the block of LD tagged by the 8 SNPs analyzed above (rs806372, rs806370, rs806369, rs1049353, rs12720071, rs806368, rs806366, rs806365). However, at least one prior report has suggested an alternate transcription start site approximately 15 kb upstream [4], [35]. We therefore also tested our traits of interest for association with an additional 14 tagging SNPs capturing the LD contained within a second haplotype block located upstream (**Figure 3**). In these analyses, total cholesterol was tested, along with LDL-C, HDL-C, logTG, and log[TG/HDL]. As shown in **Table 4**, these additional SNPs do not provide any indication of further association with lipid traits in the 15 kb upstream of rs806372.

**Table 4:** Association between median lipid traits and single SNPs, using an extended region of interest (p-values from a linear regression model in PLINK).

		Additive Mode p-value				
SNP	MAF	T CHOL	LDL-C	HDL-C	Log TG	Log (TG/HDL-C)
RS806365	0.402	0.250	0.761	0.651	0.857	0.970
RS806366	0.480	0.741	0.430	0.452	0.061	0.060
RS806368	0.202	0.437	0.124	0.204	**0.041**	**0.031**
RS12720071	0.086	**0.032**	**0.040**	0.202	0.375	0.175
RS1049353	0.312	0.159	0.527	0.707	0.994	0.860
RS806369	0.271	0.503	0.203	0.467	0.633	0.569
RS806370	0.088	0.552	0.739	0.814	0.164	0.232
RS806372	0.122	0.889	0.615	0.219	**0.020**	**0.023**
RS806376	0.499	0.814	0.091	0.872	0.857	0.861
RS806377	0.483	0.848	0.083	0.692	0.831	0.899
RS2023239	0.166	0.879	0.583	0.910	0.918	0.922
RS806379	0.479	0.874	0.249	0.898	0.716	0.737
RS6928499	0.164	0.870	0.497	0.984	0.904	0.977
RS6928813	0.164	0.870	0.483	0.974	0.930	0.962
RS9444584	0.178	0.792	0.315	0.686	0.830	0.839
RS9450898	0.164	0.874	0.462	0.928	0.944	0.973
RS7752758	0.125	0.585	0.394	0.863	0.935	0.958
RS12528858	0.066	0.842	0.882	0.083	0.449	0.198
RS12205430	0.213	0.414	0.823	0.076	0.738	0.296
RS6454673	0.302	0.842	0.425	0.342	0.893	0.599
RS6454674	0.315	0.739	0.369	0.377	0.859	0.815
RS6454676	0.107	0.248	0.151	0.916	0.975	0.858

The magnitude of effect was calculated for all variants that showed associations with lipid traits. Subjects with 0, 1 or 2 copies of the G-allele at rs12720071 have an LDL level of 117.5±27.0, 112.8±23.6, and 102.7±23.3 mg/dL, respectively. Subjects with 0, 1, or 2 copies of the C-allele at rs806368 have a mean TG level of 153.7±70.4, 163.1±74.0, and 184.3±100.9 mg/dL; and subjects with 0, 1 or 2 copies of the C-allele of rs806372 have a mean TG level of 154.1±70.7, 171.4±75.6, and 138.8±47.8 mg/dL.

To be certain that we were not missing any additional genetic association upstream, we reanalyzed these data using a more refined phenotype (i.e., modeled lipid traits, censored for co-morbidity and lipid modifying medication use) and an alternate statistical approach (i.e., non-parametric analyses using a Kruskal-Wallis test). As shown in **Table 5**, these alternate approaches failed to reveal any further association with lipids, for tagging SNPs upstream to rs806372. This approach did, however, resolve additional associations with HDL-C, located in our original region of interest (i.e., tagged by the original SNPs) [4].

**Table 5:** Association between modeled lipid trait and single SNPs, over the same extended region of interest shown in Table 4 (Kruskal-Wallis p-values are shown).

	Kruskal-Wallis p-value			
SNP	T CHOL	LDL-C	HDL-C	TG
**RS806365**	0.524	0.479	0.909	0.772
**RS806366**	0.731	0.213	0.847	0.250
**RS806368**	0.881	0.710	**0.043**	0.103
**RS12720071**	0.460	0.433	**0.029**	0.264
**RS1049353**	0.155	0.095	**0.046**	0.804
**RS806369**	0.892	0.215	0.615	0.352
**RS806370**	0.399	0.448	0.518	0.114
**RS806372**	0.076	0.375	0.218	**0.028**
**RS806376**	0.468	0.856	0.808	0.282
**RS806377**	0.480	0.671	0.962	0.491
**RS2023239**	0.846	0.207	0.192	0.906
**RS806379**	0.287	0.755	0.901	0.611
**RS6928499**	0.881	0.212	0.194	0.904
**RS6928813**	0.880	0.212	0.200	0.908
**RS9444584**	0.794	0.341	0.189	0.759
**RS9450898**	0.886	0.208	0.210	0.911
**RS7752758**	0.728	0.078	0.460	0.901
**RS12528858**	0.371	0.480	0.975	0.643
**RS12205430**	0.137	0.461	0.354	0.567
**RS6454673**	0.345	0.948	0.208	0.716
**RS6454674**	0.417	0.972	0.154	0.411
**RS6454676**	0.366	**0.040**	0.465	0.260

### Haplotype analyses

Haplotypes often provide greater statistical power than single-marker analyses, for genotype-phenotype association studies. We have previously shown that tagging SNPs capturing the linkage structure defined by physical position 88,901,306 to 88,916,775 are adequate to resolve an association with obesity-related dylipidemia [4]. Our single SNP analyses outlined above indicate that characterization of this region is sufficient to explain the entire association between CNR1 and altered lipid homeostasis. We therefore focused all subsequent analyses on the block of LD containing those specific SNPs [4]. Those 6 SNPs are highlighted in **Table 2** and **Figure 3**. It should be noted that **Tables 3** – **5** demonstrate univariate association between lipid traits and one additional SNP slightly upstream, rs806372. This SNP is in complete LD with haplotype H4, as shown below. Extending the region haplotyped to include it would therefore have been redundant.

Thus, haplotype designation followed Baye's original report [4]. Haplotype frequencies observed in the current population were again comparable to other cohorts of similar Northern European ancestry. **Table 6** demonstrates that a common CNR1 haplotype (H3, frequency 21%), is associated with fasting TG and HDL-C levels (p = 0.0307 for logTG; p = 0.0382 for HDL-C; p = 0.0038 for log[TG/HDL-C]). The results are presented in both unadjusted and adjusted format (adjusting for age, gender, BMI, physical activity and dietary fat intake). To correct for testing multiple hypotheses, we also conducted permutation testing (applying 1000 permutations) using software available in PLINK. By preserving the correlational structure between tag SNPs, this approach provides a less stringent correction than a Bonferroni correction. Because the association between H3 and obesity related dyslipidemia persists after permutation testing (empirical p = 0.0180 for log[TG/HDL-C]), it is very unlikely that these associations were observed by chance.

**Table 6:** Association between CNR1 haplotype and median lipid traits (p-values are shown, calculated using a linear regression model in PLINK).

			T Chol		LDL-C		HDL-C		Log TG		Log (TG/HDL-C)	
Haplotype†	(5′-3′) §	Freq.	Unadj.	Adj.*	Unadj.	Adj.*	Unadj.	Adj.*	Unadj.	Adj.*	Unadj.	Adj.*
H1	CCAATT	0.302	0.122	0.185	0.375	0.563	0.733	0.792	0.950	0.712	0.907	0.639
H2	CTGATC	0.267	0.766	0.659	0.315	0.187	0.277	0.471	0.864	0.878	0.428	0.778
H3	CCGATC	0.211	0.453	0.533	0.938	0.860	**0.0382**	**0.036**	**0.0307**	**0.0093**	**0.00376**	**0.00349**
H4	TCGACT	0.114	0.541	0.539	0.877	0.952	0.480	0.556	0.117	0.071	0.108	0.113
H5	CCGGCT	0.085	**0.043**	**0.036**	0.068	0.062	0.360	0.611	0.454	0.766	0.369	0.560

*adjusted by age, gender, BMI, calories from fat and sport index.

† Designation in Tes Baye paper (2008).

§ SNPs that used for haplotype construction (5′-3′): rs806370, rs806369, rs1049353, rs12720071, rs806368, rs806366.

**Table 7** suggests that these associations are clinically significant. Mean TG levels were 160±70 mg/dL, 155±70 mg/dL and 120±60 mg/dL for subjects with 0, 1, and 2 copies of the H3 haplotype. Mean HDL-C levels were 45±10 mg/dL, 47±10 mg/dL and 48±9 mg/dL, respectively. Even a modest 1 mg/dL increase in HDL-C is associated with a 6% reduction in vascular events [2]. Thus, our findings suggest that the H3 haplotype *may* exert a cardioprotective effect, a claim that warrants further study during weight gain.

**Table 7:** Effect of H3 haplocopy on lipid traits (mean ± SD).

Haplotype	Copies of Haplotype	Freq.	HDL-C (mg/dL)	TG (mg/dL)	BMI (kg/m^2^)
**H3**	0	0.63	45.9±10.4	160.1±71.7	45.2±5.1
	1	0.33	47.6±10.3	155.8±70.1	45.1±4.7
	2	0.04	48.2±9.9	120.4±61.2	44.9±4.8

## Discussion

The current study demonstrates that genetic variability in CNR1 contributes to several derangements in lipid homeostasis known to accompany weight gain. We report a CNR1 haplotype (H3, frequency 21.1%) associated with fasting TG and HDL-C levels in 645 study subjects with class III obesity, nested within one of the largest population-based biobanks in the U.S. We localize the genetic effect to a single block of LD containing the CNR1 coding region, and we demonstrate that this effect is only partly dependent upon nutrient intake and physical activity. Our findings provide evidence in support of the growing claim that CNR1 directly modulates peripheral lipid homeostasis.

### Mechanism

Endocannabinergic signaling plays a critical role in the regulation of energy metabolism. Both animal studies and clinical trials have suggested a relationship between *CNR1* and body composition [36], [37], [38]. However, studies characterizing this relationship have yielded controversial observations from different populations [39], [40], [41], [42]. One of the most well-characterized longitudinal cohort studies in the U.S, the Framingham Heart Study, recently failed to observe any association between CNR1 and obesity [42]. In retrospect, the impact of variable endocannabinergic signaling on weight gain in some cohorts may have been secondary to an underlying relationship between CNR1 and lipid homeostasis, perhaps related to an alteration in the hepatic efficiency of fatty acid beta oxidation, an alteration in fatty acid biosynthesis, or a combination of both [37].

Recently, a number of lipid and lipoprotein phenotypes have been associated with variability in *CNR1*, and the eCB signaling pathway in general [4], [7]. Our data are consistent with these reports. Endocannabinoids clearly modulate lipid traits through mechanisms other than enhanced food intake [43], [44]. For example, clinical trials conducted with CB_1_ blockers in subjects of European ancestry have revealed a greater improvement in metabolic parameters than anticipated based on weight loss alone [38], [45]. In an analysis of covariance, which corrected for weight loss through standard regression methods, patients taking CB_1_ blockers developed an increase in HDL-C and a decrease in fasting TG levels more than twice that anticipated based upon change in weight [38], [45]. Further, CNR1 knockout mice maintain a favorable lipid profile during high-fat diet [36], [37]. Altered mRNA expression in animal models and cell culture systems suggest an involvement of CNR1 in both lipogenesis and lipid transportation [46], [47], [48]. While hepatocyte-specific CNR1 knockout mice gain weight at a rate similar to their wild type littermates, they are resistant to dyslipidemia in a manner comparable with a lean phenotype [37]. While some data indicate that CB_1_ activation directly alters hepatic lipogenesis (increased acetylCoA carboxylase activity, increased fatty acid synthase activity, and decreased fatty acid β-oxidation) [37], other data suggest a role for CB_1_ in the modulation of cholesterol uptake and/or efflux [49]. The effect of CB_1_ activity on cholesterol transport may be mediated through SRB1 or ABCG1 [46]

### The role of environment

To date, common variants in biological candidate genes have only explained a small fraction (typically less than 5%) of the variance in complex traits such as dyslipidemia [50], [51]. Although it is reassuring to note that most associations identified in genome-wide scanning efforts reflect biologically plausible mechanisms, even highly heritable traits such as height (H^2^_[Height]_ ∼0.8) would require nearly 10^5^ discrete variants to explain the phenotype based on current statistical modeling [52]. Heritability for HDL-C level is robust and comparable to height (H^2^_[HDL-C]_ ∼0.7 [7]). Thus, it is plausible (and in fact likely) that gene-environment (GxE) interactions strongly influence this trait [53], [54].

For CNR1 specifically, analyses based on GxE have improved predictive power in the context of neuropsychiatric endpoints [55]. Nutrient intake is clearly a strong contributor to CNR1-environment interactions. In hepatocyte specific CNR1 knockdown animals, the metabolic effects are only present when animals are fed with a high-fat diet, not a chow diet [37]. In the current study, CNR1 interacts with dietary intake (percent calories due to fat) and vigorous physical activity (the sport index from a standardized survey instrument) to modulate lipid levels. Statistical adjustment for diet and physical activity strongly influences the association between CNR1 and TG level (as illustrated in **Table 7**), while such an adjustment has little effect on the relationship between CNR1 and HDL-C. This provides further indirect support for the claim that genetic variation in CNR1 alters fasting TG through a combination of central (appetite-related) and peripheral (lipid homeostatic) mechanisms [4], while genetic variation in CNR1 alters HDL-C level through a more direct effect localized primarily to the liver [36], [38].

### Causative variants

Overall, we observed a number of similarities between our current results, and findings reported previously in mutigenerational families of Northern European descent [4]. For example, rs806368, a SNP in the 3′-UTR of CNR1, is associated with fasting TG level in both cohorts. Further, the H4 haplotype was strongly associated with TGs in the original cohort and marginally associated with TGs in the current cohort (a cohort limited to the severely obese). Our most notable dissimilarity would be the observation that a different haplotype, H3, was associated with HDL-C in the current cohort only. Because the relationship between BMI and HDL-C is curvilinear [9], the protective effect of H3 against reduction in HDL-C level may only be evident in subjects with an extremely high BMI. It is important to note, however, that this relationship is not driven by a direct effect of CNR1 on obesity. CNR1 haplotypes were not associated with BMI in either cohort [4].

Our findings reveal that severely obese study subjects with 0, 1 and 2 copies of the H3 haplotype have mean HDL-C levels of 45±10, 47±10, and 48±9 mg/dL, respectively. Conversely, subjects with 0, 1 or 2 copies of H3 haplotype have mean TG levels of 160±70, 155±70, and 120±60 mg/dL, respectively. Although H3 appears to represent a protective haplotype (high HDL-C and low TG), the causative variants underlying the relationship between CNR1 and obesity-related dyslipidemia remain unknown. H3 is likely only in partial linkage (i.e., D'<1.0) with the causal variants, and the underlying functional alleles are probably both common and rare [52], [56]. One or more structural variants may also be contributing [57]. Repeating elements are quite common in CNR1.

It is becoming increasingly clear that the genetic control over most complex traits is due to a *combination* of common and rare variants, and that the rare variants contributing to these interactions exhibit considerably larger effect sizes than most common variants [52]. Thus, rare variants are likely to be responsible for most of the missing heritability underlying complex traits like circulating lipid levels. Future efforts must therefore be directed toward sequencing the CNR1 gene in representative subsets enriched for the traits of interest [58] and the marker(s) most strongly associated with those traits.

### Outlook

Human obesity is often co-morbid with metabolic disturbances that are ultimately more debilitating and life-threatening than obesity itself. Derangements in lipid homeostasis accompanying weight gain are due in part to genetic variation in CNR1. Efforts to re-sequence this gene in relevant cohorts will improve our understanding of dyslipidemia.
